# Supplementary material for: The Changes in the p53 Protein across the Animal Kingdom Point to Its Involvement in Longevity
Source: Int J Mol Sci. 2021 Aug 7;22(16):8512. doi: 10.3390/ijms22168512 (PMC8395165; doi:10.3390/ijms22168512)
Supplement: Supplementary file 1 [file ijms-22-08512-s001.zip › SM_02_3D structures p53 tetramers.pdf]

International Journal of *Molecular Sciences*

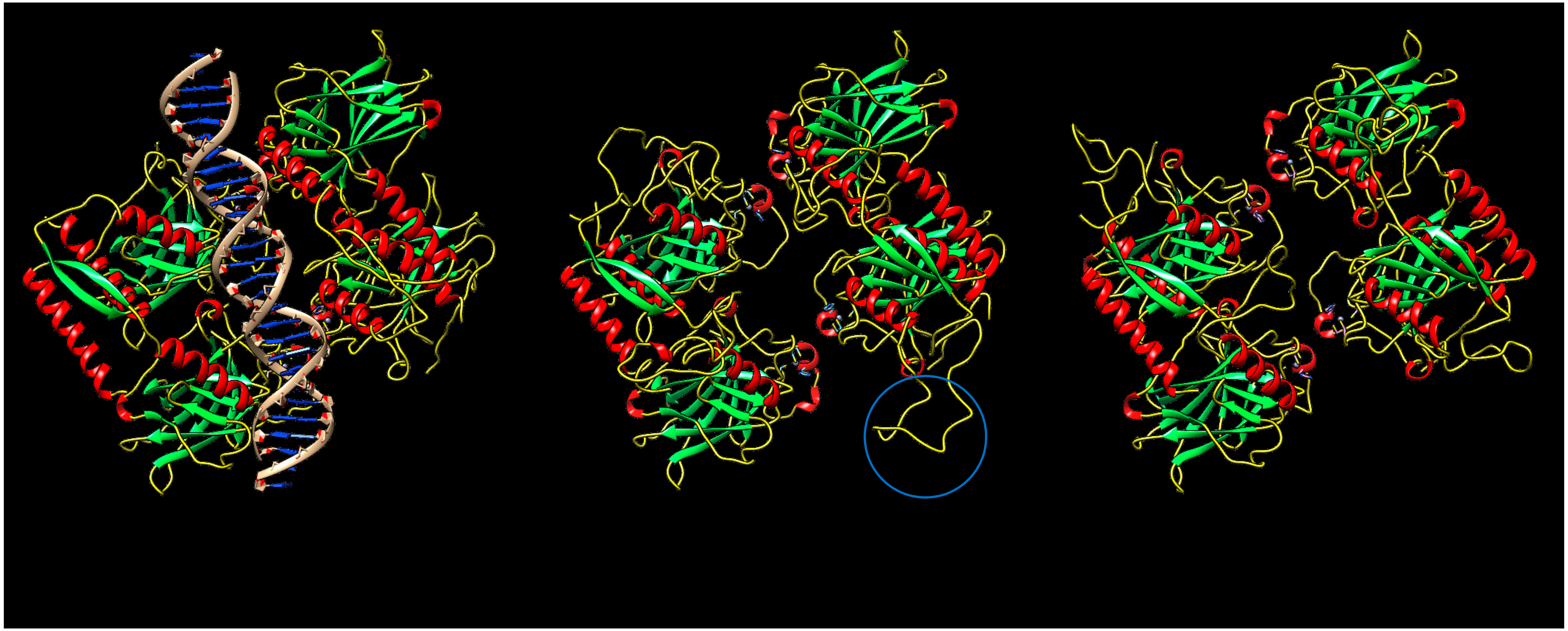

Supplementary material 2: Experimental (human) and modelled (bats) 3D structures of tetramerized p53 core domains. (Left) *Homo sapiens* p53 tetramer binding DNA (Middle) p53 tetramer from long-lived bat *Myotis Brandtii* (Right) p53 tetramer from short-lived bat *Phyllostomus discolor.* The insertion in the DNA binding domain of bats with long lifespan is present inside the DNA interaction cavity, suggesting decreased affinity of p53 for binding to DNA (blue circle).
