# Supplementary material for: The Changes in the p53 Protein across the Animal Kingdom Point to Its Involvement in Longevity
Source: Int J Mol Sci. 2021 Aug 7;22(16):8512. doi: 10.3390/ijms22168512 (PMC8395165; doi:10.3390/ijms22168512)
Supplement: Supplementary file 1 [file ijms-22-08512-s001.zip › SM_03_min_max.pdf]

International Journal of *Molecular Sciences*

MDPI

Supplementary material 3: The list of organisms with the longest and shortest lifespan in particular phylogenetic groups. The total number of analyzed animals for each group is depicted in column "Count". Formal and common names and their maximal lifespans in years are given for each organism.

Group	Count	Shortest living animal (years)	Longest living animal (years)
Afrotheria	3	*Echinops telfairi* (19)<br>Lesser hedgehog tenrec	*Trichechus manatus latirostris* (69)<br>Florida manatee
Artiodactyla	13	*Capra hircus* (20.8)<br>Goat	*Balaena mysticetus* (211)<br>Bowhead whale
Batrachia	3	*Ambystoma mexicanum* (17)<br>Axolotl	*Proteus anguinus* (102)<br>Olm
Carnivora	10	*Mustela putorius furo* (11.1)<br>Ferret	*Ursus maritimus* (43.8)<br>Polar bear
Eulipotyphla	3	*Condylura cristata* (2.5)<br>Star-nosed mole	*Erinaceus europaeus* (11.7)<br>European hedgehog
Glires	17	*Rattus norvegicus* (3.8)<br>Brown rat	*Heterocephalus glaber* (31)<br>Naked-mole rat
Chiroptera	8	*Phyllostomus discolor* (9)<br>Pale spear-nosed bat	*Myotis Brandtii* (41)<br>Brandt´s bat
Metatheria	3	*Sarcophilus harrisii* (13)<br>Tasmanian devil	*Vombatus ursinus* (30)<br>Common vombat
Neopterygii	24	*Betta splendens* (2)<br>Siamese fighting fish	*Cyprinus carpio* (47)<br>Common carp
Perissodactyla	3	*Ceratotherium simum simum* (45)<br>Southern white rhinoceros	*Equus caballus* (57)<br>Horse
Primates	15	*Carlito syrichta* (16)<br>Philippine tarsier	*Homo sapiens* (122.5)<br>Human
Sauria	13	*Anolis carolinensis* (7.2)<br>Green anole lizard	*Terrapene carolina triunguis* (138)<br>Three-toed box turtle
